# BLACK GREEK LETTER ORGANIZATIONS: FACILITATING HEALTH PROMOTION FOR AFRICAN AMERICANS ACROSS THE LIFECOURSE

**DOI:** 10.1093/geroni/igac059.1429

**Published:** 2022-12-20

**Authors:** Chivon Mingo

**Affiliations:** Georgia State University, Atlanta, Georgia, United States

## Abstract

African Americans remain underrepresented in accessing and utilizing evidenced-based health promotion interventions (EBIs). Challenges with dissemination and implementation of EBIs further corroborate existing racial/ethnic health/healthcare disparities. Therefore, there is a need to identify effective ways to increase the widespread adoption of health promotion behaviors among African Americans across the life course. It is plausible that engaging in non-traditional partnerships (i.e., community groups or organizations valued in the community with the capacity and infrastructure) could result in greater adoption and improved utilization of EBIs among African Americans. Although frequently overlooked as a study variable in empirically sound public health research, Black Greek Letter Organizations (BGLO) could be an innovative and practical approach to advancing health in the African American community. It is necessary to gain preliminary evidence of feasibility (e.g., motivation, target population reach, acceptability, ). Therefore, the purpose of this study was to conduct a content analysis to identify the intentions and communication trends of BGLOs as it pertains to public health and the African American community and assess population reach and perceptions by evaluating responses to communication specific to health promotion.We assessed health promotion patterns of four BGLOs in a ten-county metropolitan area. Coded content included communication via the organization’s webpage, Facebook, Twitter, YouTube, Instagram, and LinkedIn from a five-year time period. Findings confirm that BLGOs are invested in the health and well-being of the community, place emphasis on mitigating health inequities, and are uniquely positioned to serve as stakeholders for the translation of EBIs to end-users.

